# Multifunctional biomaterial platforms for blocking the fibrosis process and promoting cellular restoring effects in myocardial fibrosis therapy

**DOI:** 10.3389/fbioe.2022.988683

**Published:** 2022-09-15

**Authors:** Tian Yue, Shiqiang Xiong, Dezhi Zheng, Yi Wang, Pan Long, Jiali Yang, Dunzhu Danzeng, Han Gao, Xudong Wen, Xin Li, Jun Hou

**Affiliations:** ^1^ Department of Cardiology, The Affiliated Hospital of Southwest Jiaotong University, The Third People’s Hospital of Chengdu, Cardiovascular Disease Research Institute of Chengdu, Chengdu, China; ^2^ School of Life Science and Engineering, Southwest Jiaotong University, Chengdu, China; ^3^ Department of Cardiovascular Surgery, The 960th Hospital of the PLA Joint Logistic Support Force, Jinan, China; ^4^ Department of Basic Medicine, Medical College, Tibet University, Lhasa, China; ^5^ Department of Gastroenterology and Hepatology, Chengdu First People’s Hospital, Chengdu, China

**Keywords:** myocardial fibrosis, extracellular matrix, hydrogel, nanoparticles, biomaterials, multifunctional

## Abstract

Myocardial fibrosis is the result of abnormal healing after acute and chronic myocardial damage and is a direct cause of heart failure and cardiac insufficiency. The clinical approach is to preserve cardiac function and inhibit fibrosis through surgery aimed at dredging blood vessels. However, this strategy does not adequately address the deterioration of fibrosis and cardiac function recovery. Therefore, numerous biomaterial platforms have been developed to address the above issues. In this review, we summarize the existing biomaterial delivery and restoring platforms, In addition, we also clarify the therapeutic strategies based on biomaterial platforms, including general strategies to block the fibrosis process and new strategies to promote cellular restoring effects. The development of structures with the ability to block further fibrosis progression as well as to promote cardiomyocytes viability should be the main research interests in myocardial fibrosis, and the reestablishment of structures necessary for normal cardiac function is central to the treatment of myocardial fibrosis. Finally, the future application of biomaterials for myocardial fibrosis is also highlighted.

## Introduction

In recent years, the vigorous development of biomaterial technology has promoted the in-depth research in the field of precision medicine, including but not limited to drug delivery systems, implantable material andbiological imaging. (Alonzo et al., 2020; Fang et al., 2020; Hwang et al., 2017; Joanne et al., 2016; S. Liu et al., 2020; Mitchell et al., 2021; Pattar, Fatehi Hassanabad, and Fedak, 2019). Compared with systemic delivery, biomaterial delivery systems, such as nanoparticles and hydrogels, advantages more precisely in targeting to improve bioavailability and reduce side effects. (Stater, Sonay, Hart, and Grimm, 2021).Implantable materials developed using biomaterials technology, such as heart patches, vascular stents and bone defect fillers, are also partially used in clinical procedures (Joanne et al., 2016; Pattar et al., 2019; Sarig et al., 2016; Serpooshan et al., 2013; Sondermeijer et al., 2018; Xie et al., 2022; Y. Yao et al., 2022). Biomaterials have broad prospects in the medical industry. However, the worldwide focus of biomaterials on oncology related nanomedicine products have been used in the clinic (Bobo, Robinson, Islam, Thurecht, and Corrie, 2016). Biomaterials could also be used in the research of other diseases, such as cardiovascular diseases, brain diseases, and rheumatic immune diseases (Skourtis, Stavroulaki, Athanasiou, Fragouli, and Iatrou, 2020).

Myocardial fibrosis is the pathological remodeling of myocardial extracellular matrix caused by various cardiovascular diseases, which can lead to arrhythmia, heart failure, and even sudden cardiac arrest and sudden cardiac death. It is the common pathological mechanism of most cardiovascular diseases (such as myocardial infarction, hypertension, hypertrophic cardiomyopathy, myocarditis, aortic stenosis, etc.), and its continuous progress will eventually lead to adverse clinical outcomes such as heart failure and death (F. Yu, McLean, Badiwala, and Billia, 2022; Ziaeian and Fonarow, 2016). Studies on the pathogenesis of myocardial fibrosis have found that it is closely related to renin angiotensin aldosterone system, regulatory cytokines, non-coding RNAs, oxidative stress, matrix metalloproteinase system, endothelial dysfunction, immune inflammatory response and so on. (Karsdal et al., 2017; Sumners, Peluso, Haugaard, Bertelsen, and Steckelings, 2019; Pinar et al., 2020; Yokota et al., 2020; Passaro et al., 2021).

In this review, we focus on recent research advance in biomaterials-based on therapeutic strategies for myocardial fibrosis, and summarize different types of biomaterials used in the treatment of myocardial fibrosis such as injectable hydrogels, patches and nanoparticles to block myocardial fibrosis processes and restore cardiomyocytes viability.

## Pathology of myocardial fibrosis

Myocardial fibrosis, also known as myocardial calcification, is formed by an abnormal healing process after myocardial cell injury, eventually leading to the end-stage of extracellular matrix (ECM) remodeling and heart failure(HF)(Lopez et al., 2021; Tsao et al., 2022).

In continuous or acute ischemia and hypoxia, myocardial infarction (MI) and reperfusion injury (IR), such as myocardial necrosis and atherosclerotic plaque rupture caused by coronary artery disease, both of them will lead to a series of ECM remodeling and fibrosis (Libby et al., 2019; Y. Yao et al., 2022). There are generally three stages of myocardial recovery after MI. Firstly, the short-term necrotic phase after myocardial infarction, is characterized by high oxidative stress. At this stage, apoptotic cardiomyocytes rupture and release large amounts of cellular contents to the ECM, as well as a large amount of reactive oxygen species (ROS), which is one of the key substances in the early stages of infarction (Maxwell, 1999; Ruparelia, Chai, Fisher, and Choudhury, 2017). Second, the inflammatory stage, is divided into the acute inflammatory stage and chronic inflammatory stage. The acute inflammatory phase is characterized by prominent levels of pro-inflammatory factors and immune cells recruitment. The explosive levels of ROS released during the necrotic, as well as cellular contents, stimulate the onset of inflammation and lead to the polarization of macrophages toward M1 (pro-inflammatory type)(Y. Yao et al., 2022). In this stage, matrix metalloproteinase (MMP), secreted by inflammatory cells, degrade the natural ECM, and cause ventricular thinning and left ventricular (LV) remodeling (Carlini et al., 2019). Finally, with the removal of leakage contents, the body initiates a chronic inflammatory repair stage. Inflammatory cells release transforming growth factor-β (TGF-β) and increase the production of metalloproteinase inhibitors (TIMPs), so as to reduce enzymatic degradation in injury, promote cell proliferation, migration, differentiation, accelerate granulation tissue formation, and promote wound healing for cardiomyocyte transformation and repair (J. Chen et al., 2018). Unfortunately, TGF-β has no expected effect on myocardial recovery and repair, as a key factor in myocardial fibrosis, TGF-β promotes the activation of cardiac fibroblasts and facilitates the conversion of cardiac fibroblasts to myofibroblasts while prompting the secretion of type I and III collagen and reducing collagenase synthesis, leading to excessive deposition of collagen and eventual fibrosis (Darby, Laverdet, Bonté, and Desmoulière, 2014; Yokota et al., 2020).

Chronic myocardial fibrosis, also known as diffuse myocardial fibrosis, is usually caused by cardiac overload or drug injury (Lopez et al., 2021). Cardiac overload includes pressure overload or volume overload, of myocardial inflammation (myocarditis), metabolic disease (obesity and diabetes), aging, and drug-induced fibrosis (adriamycin myocardial injury)(Aminian et al., 2021; Ammirati et al., 2021; Gazoti Debessa et al., 2001; Homsi et al., 2017; Mannacio et al., 2015; Ritchie and Abel, 2020; S. Zhang et al., 2012). Diffuse myocardial fibrosis is also accompanied by chronic inflammation in which cardiac macrophages are activated and cardiac fibroblasts are gradually activated (Hulsmans et al., 2018). NLRP3 inflammasome and TGF-β are produced in immune cells, endothelial cells, cardiomyocytes and cardiac fibroblasts (Hanna and Frangogiannis, 2019; Pinar et al., 2020). In addition, unlike acute myocardial infarction, hypoxic injury of cardiomyocytes is accompanied by mitochondrial dysfunction, leading to the disruption of the balance of ROS production and cleareance and the increase of ROS (Li X et al., 2020). Endoplasmic reticulum stress (ERS) and activation of unfolded protein can also stimulate fibrosis, which promotes epithelial-mesenchymal transition and stimulates inflammation(Liu et al., 2021; Lopez et al., 2021).

### The process of myocardial fibrosis

**FIGURE 1:**
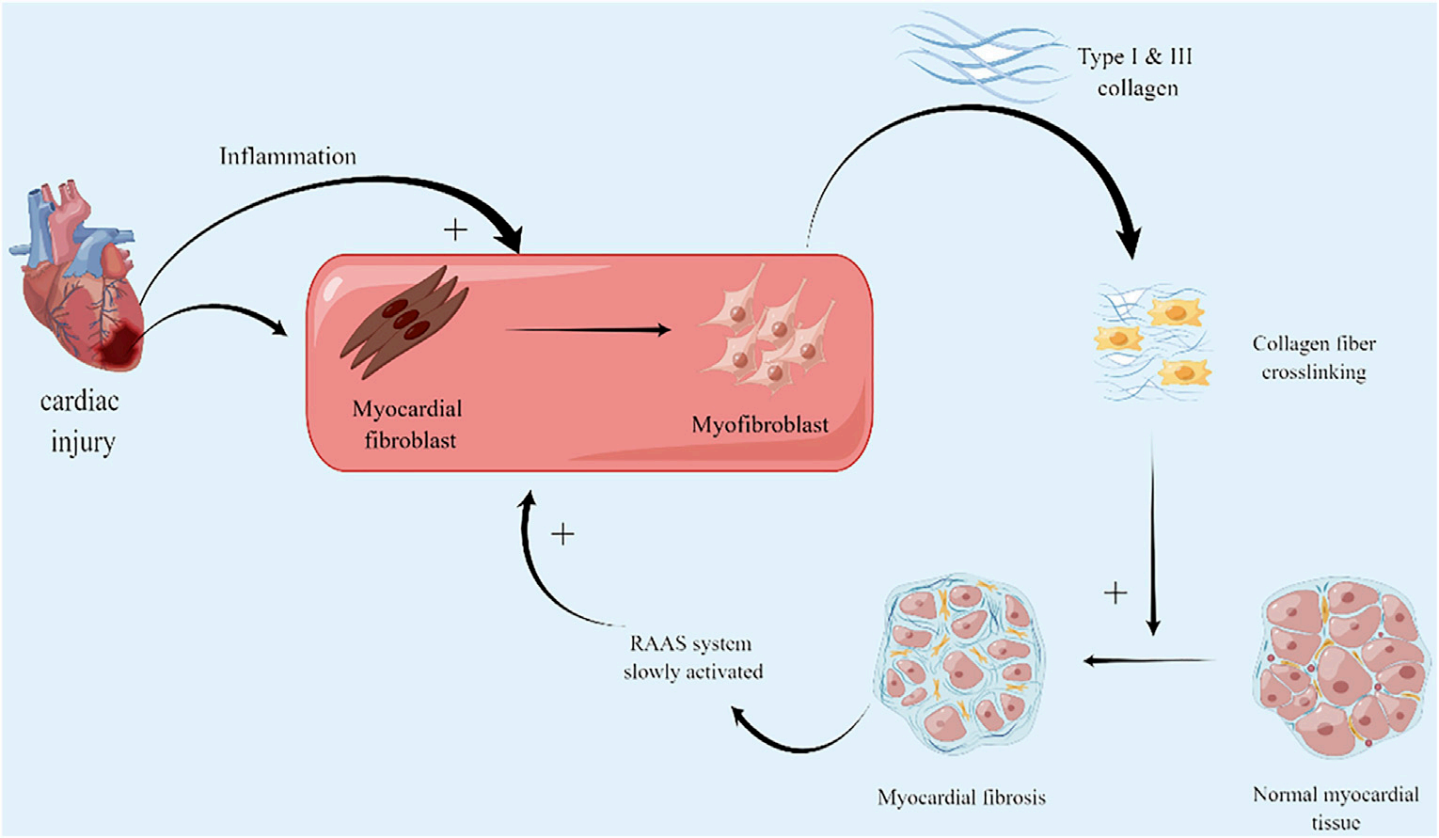
Myocardial fibrosis mechanism: firstly, activation and differentiation of myocardial fibroblasts after myocardial injury. Excessive deposition of type I and III collagen leads to fibrosis; secondly, RAAS stimulated by fibrotic mechanical stress, increases the burden on the heart, leading to further myocardial damage and aggravingfibrosis. (By Figdraw).

In diffuse fibrosis, myocardial fibroblasts play a crucial role in the pathological process. Myocardial fibroblasts can be derived from the myocardium itself or cell transformation such as fibroblasts and epicardial epithelial cells (Furtado, Costa, and Rosenthal, 2016). The altered biological and mechanical stresses caused by myocardial injury lead to the differentiation of activated cardiac fibroblasts in ECM into myofibroblasts. The activation of myocardial fibroblasts promote the increase of fibrillar collagen synthesis, i.e., the increase of synthesis of type I and type III collagen and excessive deposition in ECM (Herpel et al., 2006). However, the degradation of fibrillar collagen does not alter or increase, which disrupts the normal physiological structure of the myocardium and increases the stiffness to a certain extent thus losing the myocardial elasticity that ensures normal cardiac function (Figure 1)(Yokota et al., 2020). The exploration of pathological processes of myocardial fibrosis is gradually maturing. Both acute and chronic fibrosis processes are accompanied by myocardial cell injury, chronic activation of inflammation, activation of the RAAS system, and promotion of myocardial fibroblasts and myofibroblasts. This process might be the mechanism of the internal protection and repair of the body. However, the activation of inflammatory and repair processes and the scarring of the protective cardiac structures did not eventually end at the right stage. These processes stimulate each other and continue to circulate, leading to more serious fibrosis, further loss of cardiac function, and ultimately HF.

## Biomaterial-based therapeutic strategies

According to the 2021 European Society of Cardiology (ESC) guidelines for the treatment of acute and chronic HF, myocardial ischemic injury caused by acute myocardial infarction is currently treated by surgery such as bypass and stent implantation, with the primary purpose of dredging the blood flow (McDonagh et al., 2021). Percutaneous coronary intervention (PCI) using thrombolytic agents and coronary artery bypass grafting (CABG) is the best way to restore blood and oxygen supply after MI. HF can be treated with a left ventricular assist devices or heart transplant (Lamour et al., 2009; Mancini and Colombo, 2015). In addition, angiotensin-converting enzyme inhibitors (ACEI) or angiotensin receptor enkephalins inhibitors (ARNI), beta-blockers, Sal corticoid receptor antagonists (MRA) and sympathetic blockade can reduce the risk of HF patient. However, these treatments merely slow down the process of HF and fibrosis without restoring cardiac function (McDonagh et al., 2021). Therefore, the research strategies for cardiac function injury need to shift the focus on how to reduce further damage and explore achievement on restoration of cardiac function. Based on the pathological process of fibrosis, combined with functional biomaterials, inhibiting chronic inflammation, suppressing slow activation of RAAS, and reconstructing ECM environment have become a promising research direction for myocardial fibrosis (Hayashi, Takai, and Yamashita, 2010; Sager et al., 2016; Wang L et al., 2021).

### General strategies

#### Therapeutic strategies for ECM

ECM isa complex framework constructed by a various biomolecule around cells to maintain the natural physiological function of myocardiocytes and the physiological structure of the heart (Wang X et al., 2021). The activation of inflammation leads to the release of (MMP), which leads to the degradation and remodeling of ECM. Subsequently, collagen is continuously deposited in the ECM, and necrotic cardiac tissue is permanently replaced by fibrotic ECM. On the contrary, the restoration of normal physiological function and structure of ECM has now been shown to be beneficial to myocardial fibrosis (X. Wang et al., 2022). At present, biomaterials are currently applied to restore the ECM with functions including re-establishing microenvironmental blood flow, providing mechanical support, softening the fibrotic ECM, and protecting cardiomyocyte (Y. Li et al., 2021; Ozbek, Balasubramanian, Chiquet-Ehrismann, Tucker, and Adams, 2010; Rodell et al., 2016).

### Blood supply reconstruction

After myocardial injury, the body initiates its vascular repair process while this process cannot meet the needs of the residual ischemic cardiomyocytes (Awada et al., 2017). Cytokines, chemokines, polysaccharides, and nucleic acids can achieve angiogenesis in a short period to restore blood supply. Vascular endothelial-derived growth factor (VEGF) is a cytokine that plays a key role in homeostasis and vascular regulation in the process of disease *in vivo*. In tumor tissue, it can significantly trigger cardiovascular formation and contribute to the growth of tumor tissue (Apte, Chen, and Ferrara, 2019). On the contrary, in myocardial ischemia, angiogenesis induced by VEGF can enable blood supply reconstruction and restore blood supply to cardiomyocytes. Due to its dual nature, the application of VEGF requires attention to precise delivery and controlled release (Tsutsumi and Losordo, 2005). In addition, platelet-derived growth factor (PDGF), hepatocyte growth factor (HGF), and angiopoietin-1 (ANG-1) can also affect the proliferation and differentiation of vascular endothelial cells (Borrelli, Turnquist, and Little, 2021). Basic fibroblast growth factor-2 (FGF-2) can also promote cardiovascular growth by upregulating VEGF (Awada et al., 2017). In recent studies, the synthetic peptide Arg-Glu-Asp-Val (REDV) can promote vascular growth and it is easier to use a biological delivery systems to deliver in the form of packaging (Y. Yao et al., 2022). Although cytokines act directly on blood vessels, chemokines promote angiogenesis by recruiting stem cells. For example, HDAC7, which belongs to the class II HDAC family, is specifically expressed in vascular endothelium and increases migration, proliferation and differentiation of stem cells, especially targeting vascular progenitor cells (VPC)(You et al., 2021). Stromal cell-derived factor 1α (SDF-1α) is an effective chemokine that can recruit stem cells to the infarcted area to achieve myocardial repair (Awada et al., 2017). In addition to proteins, nucleic acids can be used as an effective biomolecule for myocardial injury to re-establish blood supply. microRNA-21-5p (miR-21-5p) is highly expressed in endothelial cells and stimulates angiogenesis by targeting anti-angiogenic genes(Y. Li et al., 2021). Several components of ECM can be used to promote angiogenesis in injured tissues. Hyaluronic acid (HA) is a natural polysaccharide and a key component of ECM, plays a crucial role in biological processes, including the promotion of vascular growth and inflammation (Jiang, Liang, and Noble, 2007).

### Mechanical support and scar softening

Among biomaterials used for cardiomyocyte functional protection, tissue-engineered patches and injectable hydrogels can provide mechanical support and soften scarring (Ogle et al., 2016). The mechanical support and scar softening effect of materials are drived from the interlinked chemical structure of the material, which is generally a functional characteristic of the biomaterial itself and does not require the assistance of active pharmaceutical ingredients (Le et al., 2018). Most of these materials are modified biomaterials derived partially or entirely from the ECM. HA, chitosan, peptides, collagen, etc. can be used as synthetic raw materials for injectable hydrogel and patch materials to provide mechanical support (Carlini et al., 2019; He et al., 2020; Le et al., 2018; Y. Zhang et al., 2019). Among these, type I collagen can be used as a source of injectable biomaterials, but since the excessive deposition of type I collagen is inherently accompanied by the fibrosis process, it is still worth considering whether the use of type I collagen as a support material is beneficial (Y. Zhang et al., 2019). In addition, homologous decellularized extracellular matrix (dECM) is also a supplement for ECM. It not only provides support, but also contains a variety of cytokines and chemokines, providing a good healing environment for injured tissues (Ozbek et al., 2010).

### Cardiomyocyte protection

Injury to cardiomyocytes is the beginning of fibrosis, and local inflammation after cardiomyocyte injury is accompanied by the release of a large variety of bioactive proteins, which may lead to the destruction of ECM stability and is detrimental to the maintenance of the normal function of the remaining cardiomyocytes (Domenge et al., 2021). There are many signaling pathways that are expected to be targets for inhibiting cardiomyocytes apoptosis. Take insulin-like growth factor-1 (IGF-I) as an example, which activates the PI3K/Akt pathway, plays a crucial role in cell survival, achieves potent cardioprotective and anti-apoptotic factor effects (Suleiman, Singh, and Stewart, 2007). Apoptosis-related signaling pathways such as the Ras-Raf-MEK-ERK pathway and the ROCK pathway can also achieve myocardial protection (Brown, Fiore, Sulchek, and Barker, 2013; Y. Wang, 2007)

### Targeting inflammations

The recovery process after cellular injury is accompanied by complex inflammation and immune response. Inhibition of related cytokines, inflammatory factors and immune cells has been proved to effectively block the fibrosis process (Mack, 2018). Some common inflammation inhibitors, such as colchicine and tacrolimus have been shown to be effective in inhibiting fibrosis (Okuda et al., 2016; Wang L et al., 2021). In addition to traditional small molecule drugs, targeting the key mediators in the process of inflammatory-fibrosis is also a therapeutic strategy. The NLRP3 inflammasomes is a multiprotein oligomeric complex responsible to produce interleukin-1β (IL-1β) and IL-18 to activate the inflammatory response. The NLRP3 is a promoter of the initiation of the cardiovascular healing and tissue scar formation. The development of drugs targeting NLRP3 can be used as an auxiliary to inhibit the pro fibrotic process caused by inflammation (Pinar et al., 2020). Some common inflammatory mediators also have pro-fibrotic effects such as IL-1, tumor necrosis factor-α (TNF-α), IL-6, IL-8, and IL-12, which may be regarded as potential therapeutic targets for myocardial fibrosis (Mack, 2018).

In terms of cells, the homeostatic state of helper T-cell 1 (Th-1) and T-cell 2 (Th-2) cells has a significant effect on myocardial fibrosis after injury. Cytokines released by Th-2 cells, such as IL-4, IL-5, IL-10, and IL-13, are powerful fibro genetic factors. Among them, IL-4, IL-5, and IL-13 are associated with the development of fibrosis. IL-4 activates B cells, induces initial CD4^+^ T cell differentiation to the Th-2 phenotype, and promotes the development of M2 type monocytes/macrophages (Choy et al., 2015; Mack, 2018). M1/M2 macrophage balance can also affect myocardial fibrosis, since M1 macrophages induce a severe inflammatory response while M2 macrophage secrete IL-4, IL-10, IL-13 and transforming growth factor-beta (TGF-β), all of these would aggravate the degree of fibrosis (Mack, 2018).Using immune tolerance mechanisms to induce specific immune tolerance to cardiomyocyte antigens is also a strategy for long-term strategy. Immune tolerance is induced by the combination of antigen and immunosuppressant (such as rapamycin). This process induces antigen-specific regulatory T cells (Treg), which mediate immune tolerance and alleviate inflammation, suppress toxic T cells and the phenotype of M1 macrophages (van der Laan, Nahrendorf, and Piek, 2012; Saxena et al., 2014; Kwon et al., 2021). However, as mentioned above, in the long run, the balance of M1/M2 macrophages is conducive to the pathological process of fibrosis. Thus, specific immune tolerance is a new therapeutic strategy using immunity and inflammation, but this strategy may need some optimizations.

The inflammation process involves multiple cytokines, inflammatory factors, and immune cells. It is generally believed that inhibiting inflammation can reduce fibrosis. However, several studies suggest that inflammation is not a prerequisite for tissue regeneration and does not necessarily lead to fibrosis (Mack, 2018). Therefore, our strategy for treating myocardial fibrosis after injury should shift from overall inhibition to balance regulation. The healing process requires a dynamic and stable environment to restore the damaged myocardium rather than completely inhibiting signals or factors.

### Targeting the renin-angiotensin-aldosterone system (RAAS)

**FIGURE 2:**
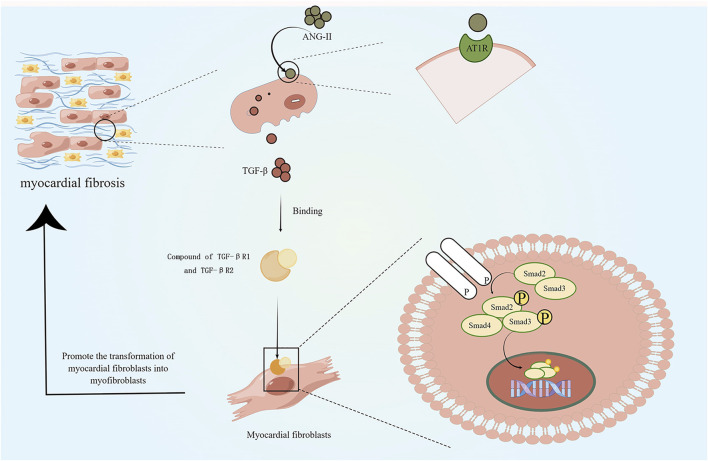
Effects of slow activation of RAAS on myocardial fibrosis: slow activation of RAAS leads to release of angiotensin II, which stimulates the release of transforming growth factor-β (TGF-β) via the AT1R pathway. TGF-βR1 forms a complex with TGF-βR2 and stimulates downward phosphorylation of the intracellular effector protein Smad2/3, which forms with Smad4 complex and moves to the nucleus. This process contributes to the differentiation of cardiac fibroblasts into myofibroblasts and increases collagen deposition. (By Figdraw).

The stimulation of myocardial fibrosis usually leads to the slow activation of RAAS, which further increases the load on the injured heart and leads to the further increase in myocardial fibrosis as well (Figure 2). Inhibiting the slow activation of RAAS system or reducing the effects of angiotensin II (ANG-II) is beneficial to the injured heart and can slow down the process of HF (Hayashi et al., 2010; AlQudah, Hale, and Czubryt, 2020). ANG-II receptor antagonists are used to reduce the cardiac overload associated with RAAS activation. However, in recent years, with the development of biomaterial delivery platforms, the role of recombinant human angiotensin-converting enzyme 2 (rhACE2) in cardiovascular has attracted more and more attention. rhACE2 is a novel ANG-II negative regulator that cleaves ANG-II and produces cardioprotective 7-peptide (ANG1-7)(Qiu et al., 2021). The ACE-II is also a major downstream molecule of catestatin, which has multiple cardioprotective effects. In clinical settings, ACE-2 has also been shown to restore ANG-II-induced myocardial fibrosis, oxidative damage, and cardiac dysfunction. (Qiu et al., 2021). Angiotensin as a therapeutic target may reduce further deterioration of fibrosis caused by RAAS activation, but it cannot be used as a therapeutic strategy to restore cardiac function. The combination of RAAS inhibition and other therapeutic strategies with myocardial restoration may be an ideal direction.

### Treatment strategies with myocardial repair effects

#### Cellular therapy

**FIGURE 3:**
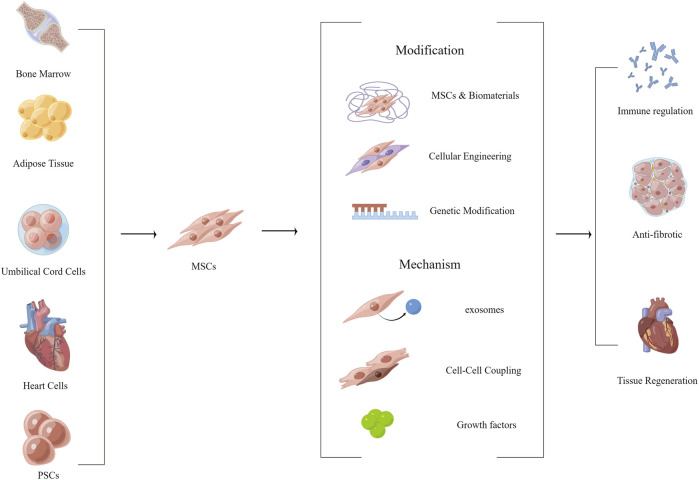
Functionalized modification of MSCs, effect and mechanism of MSCs on myocardial fibrosis. (By Figdraw).

Intramyocardial or epicardial transplantation of cardiomyocytes or stem cells can maintain or even restore cardiac function in a paracrine like manner, or can restore lost cell populations (Hatzistergos et al., 2010; Bargehr et al., 2019). Previous studies have demonstrated that cardiomyocyte-derived cells (CDCs), induced pluripotent stem cells (iPSCs), embryonic cells, endothelial progenitor cells (EPCs), and mesenchymal stem cells (MSCs) have a positive effect on the repair of damaged myocardium (Bierie et al., 2009; J. Chen et al., 2018; Kisseleva and Brenner, 2021; Laflamme et al., 2007; Pittenger and Martin, 2004). PSCs may differentiate into all types of somatic cells and subsequently repair multiple tissues (Weiskirchen, Weiskirchen, and Tacke, 2019). Both adipose-derived MSCs and amniotic-derived MSCs were utilized for repairing myocardial injury (Y. Chen et al., 2020; You et al., 2021). MSCs can consistently produce and release therapeutic molecules that control apoptosis, inflammatory response through paracrine signaling, accompanied by cytokine upregulation such as HIF1-α, IL-10, TIMP-1, MMP-2, IGF-1, CXC12 and FGF-2, which can realize inhibition of inflammation, inhibit extracellular matrix remodeling, and achieve blood supply reconstitution (Figure 3)(Gnecchi et al., 2006; You et al., 2021). Alternatively, stem cell-related technologies can be used to improve the therapeutic effect of stem cells and repair lost cell populations to a certain extent through direct programming or paracrine signaling by cell injection (Awada et al., 2017). Previously, MSCs were often delivered by direct injection, such as intracardiac, intravenous, or coronary catheter delivery (Melhem et al., 2017). However, such an injection method suffers from low retention rates of cell transplantation and poor long-term cell survival (Zeng et al., 2007). The use of injectable hydrogels or cardiac patches allows for excellent delivery of cells and minimizes cell displacement. It also provides a normal growth environment for stem cells and maintains cell viability and secretory activity (Mirotsou, Jayawardena, Schmeckpeper, Gnecchi, and Dzau, 2011; Chi et al., 2012; Mewhort, Turnbull, Meijndert, Ngu, and Fedak, 2014). Stem cell therapy is one of the current promising methods to repair partially missing myocardium, capable of achieving a variety of beneficial cardiomyocyte and ECM functions through paracrine like secretion. However, the excessive cost and huge risk of transplantation make people doubt its effectiveness and safety, and regulatory agencies have strict requirements for clinical trials of stem cells (J. Tang et al., 2022). Studies have also demonstrated that the tumorigenic risk, immune risk, and off-target effect of stem cell therapy existed (Heslop et al., 2015; J. N. Tang et al., 2018). Therefore, there are still many problems to be solved in stem cell therapy, but this is not a bad direction.

#### MicroRNA

In recent years, with the continuous development of microRNA (miRNA) technology using miRNA as a target or miRNA as a therapeutic active ingredient to regulate cellular protein expression to treat myocardial fibrosis has achieved initial success. Researchers used CDR132(an antisense oligonucleotide) to specifically inhibit the function of miR-132, normalize cardiomyocytes, and prevent and reverse the pathological remodeling process of the myocardium. This is the first miRNA drug proved to be safe and effective in the treatment of HF. This study is also a milestone breakthrough in the application of miR in HF treatment (Nicholls, 2022). In addition, miRNAs can simultaneously promote the secretion of a variety of endogenous molecules which regulate various aspects of angiogenesis, inflammation, and cell differentiation (Y. Li et al., 2021). Several anti-apoptotic miRs, such as miR-24 and miR-214,related to cell proliferation, miR-19 and 590, and miR-210 related to cardiovascular generation, have been shown to be beneficial to myocardial recovery (J. Chen et al., 2013; Eulalio et al., 2012; Harel-Adar et al., 2011; Hu et al., 2010; Verma, 2013). However, the use of miRNAs for therapeutic purposes requires attention to the fact that exposed miRNAs need to be protected by encapsulation to prevent failure caused by cyclic degradation (Y. Li et al., 2021). Besides, miRNAs are required to enter the cell to perform their functions, so the delivery platform for miRNAs needs to be modified to increase cellular uptake (Bheri and Davis, 2019). Generally, miRNAs are negatively charged and can be bonded with biological materials for intracardiac delivery using electrostatic adsorption (Y. Li et al., 2021). Nucleic acids can modify the composition of local signaling molecules and enhance repair and regeneration, making it an ideal choice for effective biomolecules in myocardial injury (Sager et al., 2016). The mechanisms of miRNA therapies are more clearly defined than that of stem cell therapies. However, it is still worth considering whether therapeutic modalities that regulate the expression of a single gene to cope with the complex physiological environment could continue to be optimized.

#### Exosome therapy

**FIGURE 4:**
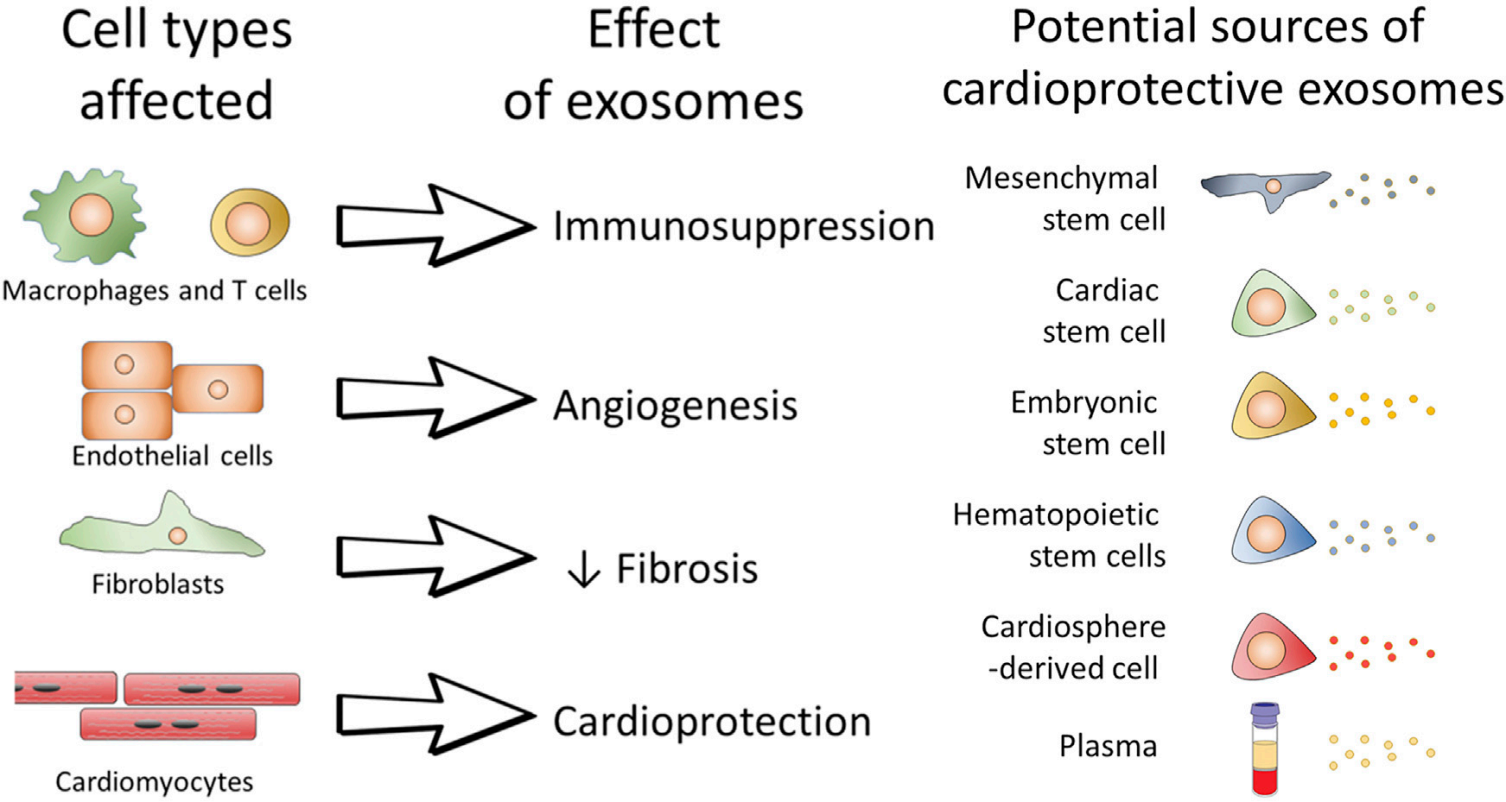
The role of exosomes on individual cells and the possible potential therapeutic role of exosomes.(Davidson & Yellon, 2018)

Exosomes are nano-vesicles secreted by cells, which can encapsulate nucleic acids, proteins, lipids, amino acids, and other metabolites. As a “messengers” of proximal intercellular communication, exosomes can affect cells in both healthy and diseased states (Figure 4)(Kalluri and LeBleu, 2020). Cardiovascular exosomes are secreted by mesenchymal stem cells, pluripotent stem cells, cardiomyocyte-derived cells, etc. (Gao et al., 2020; Nguyen, Dawkins, Bi, Marbán, and Li, 2018; J. Tang et al., 2022). Exosomes derived from cardio sphere-derived cells (CDC) improve poor myocardial remodeling, inhibit fibrosis, and improve post-infarction cardiac function in pigs (Gallet et al., 2017). Reports have demonstrated that iPSC-derived exosomes can act on cardiomyocytes to reduce apoptosis, maintain calcium stability and promote vascular growth (Gao et al., 2020). In addition, some exosomes can also affect macrophages and regulate the polarization of macrophages to reduce the inflammatory response (Hu et al., 2021). Like cells, exosomes delivered to the heart degrade in a short time and cannot be retained in injuried area to obtain sustained effects. Therefore, the use of exosomes as therapeutic agents also requires consideration of the targeting, local retention capacity and biological activity of the inclusions (J. Yao et al., 2021). It is undeniable that exogenous therapy is conducive to the recovery of cardiomyocytes. However, exosomes are of diverse origin and contain complex contents, and are basically tools for intercellular communication. The specific effects of exosome regulation is influenced by the cells that secrete them (Kalluri and LeBleu, 2020). Due to the limitations of acquisition, preservation, transportation and safety, there are still many problems in clinical applications.

## Biomaterials in myocardial fibrosis studies and therapy

Aiming at the pathological process of myocardial fibrosis, the current research on the treatment of myocardial fibrosis mainly focus on the inhibition of inflammation, the modulation of RAAS, the promotion of local microvascular revascularization, the regulation and support of ECM and stem cell therapy. In the case of therapeutic strategies that target the above processes, most delivery agents, such as colchicine or tetracyclic immunosuppressants, cytokines, chemokines, miRNAs, etc., need to realize local microenvironmental delivery to achieve effective utilization of drugs to perform therapeutic effects, or only local targeted delivery can mitigate the side effects of the drugs. Therefore, it is very important to use biomaterial drug delivery system for myocardial fibrosis drug delivery.

### Hydrogel

Many acute or chronic myocytes injuries and deficiencies can trigger or further aggravate myocardial fibrosis. In acute myocardial injury, such as MI and reperfusion injury, myocardial fibrosis is accompanied by acute inflammation, leading to ECM degradation and remodeling, insufficient blood supply of local microenvironment and excessive collagen deposition in a short time (Y. Li et al., 2021). The increase of ventricular wall hardness is caused by the excessive deposition of collagen of type I and III. At the same time, mechanical stress and the further deposition of collagen slowly stimulate the activation and transformation of myocardial fibroblasts, leading to a vicious cycle of aggravated fibrosis (Awada et al., 2017). The pathological process of acute MI also provides us with ideas for the treatment of fibrosis caused by acute MI, i.e., to provide mechanical support for the infarcted area (Awada et al., 2017), and gel provides an environment for myocardial cells to maintain normal function (Bao et al., 2017; C. Fan et al., 2019; Liu Y et al., 2021). Softening of over deposited proteins reduces excessive activation and transformation of cardiac fibroblasts caused by mechanical stress stimulation and stretch (Domenge et al., 2021). Based on this, researchers delivered active peptides/proteins, stem cells, etc. to re-establish blood supply and replenish missing cells (Y. Chen et al., 2020; You et al., 2021; Y. Zhang et al., 2019). Hydrogel is a hydrophilic polymer material with a crosslinked spatial network structure (Q. Huang et al., 2017). In cardiovascular disease, hydrogels have many advantages, such as cardiovascular structure, simulating ECM environment, biodegradability, and *in-situ* transfer of active elements. (Y. Chen et al., 2020; Domenge et al., 2021; Y. Zhang et al., 2019; Zheng et al., 2021). Injectable hydrogels can be derived from a variety of materials, including natural gel materials such as Homogeneous or heterogeneous ECM (X. Wang et al., 2022; Wang X et al., 2021), alginate (J. Yu et al., 2009), HA (Dorsey et al., 2015; Y. Li et al., 2021), collagens (C. Fan et al., 2019), fibrous protein (Losi et al., 2010; Awada et al., 2017), heparin (Prokoph et al., 2012), and chitosan, etc. (Domenge et al., 2021; H. Wang et al., 2010).and synthetic polymeric gel materials (Carlini et al., 2019), . such as Poly(2-alkyl-2-oxazoline) (POx) derivatives of 2-ethyl-2-oxazoline and 2-butenyl-2-oxazoline (He et al., 2020), Poly(N-isopropylacrylamide) nanogel (FSN)(Mihalko, Huang, Sproul, Cheng, and Brown, 2018). Natural gels can also be used in combination with artificial polymeric gels, such as polypyrrole-chitosan hydrogels (PPY-CHI)(He et al., 2020)

#### Hydrogels of natural origin

In addition to providing mechanical support, some natural gels have their own functions, and most of which come from ECM itself or materials with ECM-like composition (Table 1)(Y. Chen et al., 2020). Such functional materials are used to deliver effective molecules with therapeutic effects, and can simply achieve various therapeutic effects (Le et al., 2018). ECM of human- and porcine-derived cardiomyocytes has been used for the treatment of fibrosis after MI. The decellularized extracellular matrix (dECM), obtained by decellularization is rich in biologic factors, which can provide a cellular growth environment, softenten the microenvironment, maintain the normal function of normal cardiomyocytes, reduce the conversion of cardiac fibroblasts to myofibroblasts, decrease the expression of transforming growth factor-β (TGF-β), and reduce matrix collagen deposition (X. Wang et al., 2022; Wang X et al., 2021). Using natural gel to treat cardiomyocyte injury is theoretically very promising, but there are still some problems. Natural gels usually contain large amounts of peptides/proteins. Take dECM as an example, whether dECM of heterologous source tends lead to severe local immune response and contribute to further deterioration. However, due to the limitations of theory, cost and scope of application, there are also many problems that need to be solved before human derived dECM can be used in clinical treatment (Wang X et al., 2021)

**TABLE 1:** Naturally sourced gel materials.

Category	Target	Materials	Features	References
Natural	ECM	Fibrin Gel	Step by step release and slow release	Awada et al., 2017
		Collagen I hydrogel	Supportability	(C. Fan et al., 2019; Y. Zhang et al., 2019)
		Chitosan	Enhanced absorption	Domenge et al., 2021
	Cells	Elastin mimetic peptide hydrogel (EMH)	Self-repair and self-assembly	(R. Chen et al., 2021)
		Porcine acellular extracellular matrix (dECM)	Provide cell growth environment	(X. Wang et al., 2022)
		Human acellular extracellular matrix	Provide cell growth environment	Wang X et al., 2021
	inflammation	Hyaluronic acid (HA)	anti-inflammatory	Le et al., 2018

#### Synthetic hydrogel

**FIGURE 5:**
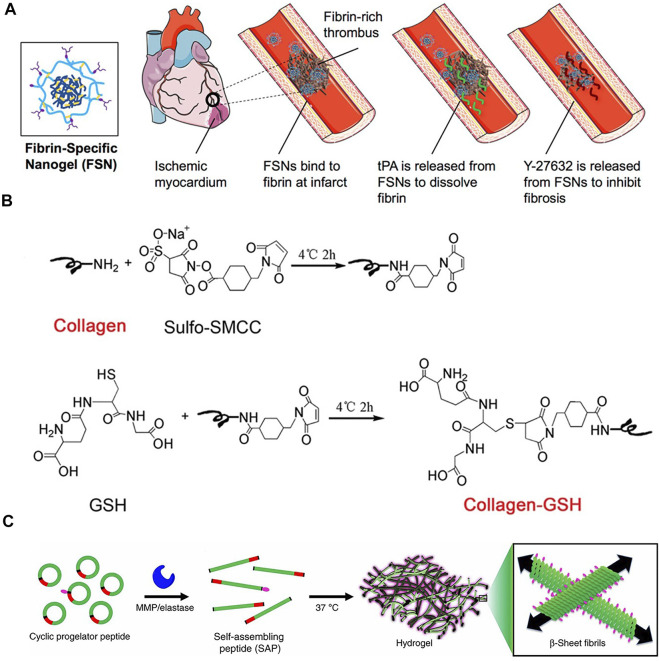
**(A)**Fibronectin-specific poly (N-isopropylacrylamide) nanogels (FSN)(Mihalko et al., 2018) **(B)** A schematic of GSH conjugation on collagen using Sulfo-SMCC.(C. Fan et al., 2019) **(C)** A cyclic pre-gelatin peptide containing a gelation sequence (green), a matrix metalloproteinase (MMP)/elastase cleavage recognition sequence (red) and a disulfide bond (black) that resists assembly due to conformational restrictions(Carlini et al., 2019).

Synthetic hydrogels for *in vivo* injection are usually synthesized from metabolizable materials (Table 2) and modified with hydrophilic groups to increase the void space of the gel to increase the drug loading capacity. Carboxyl groups such as polylactic acid and polyvinyl alcohol can increase hydrophilicity and degraded into monomers such as lactic acid, which can metabolize into carbon dioxide and water for excretion (Zheng et al., 2021). Some synthetic proteins, such as elastin mimetic peptide hydrogel (EMH) and type I collagen hydrogel, can metabolize normally and non-toxically *in vivo*. Due to the self-assembly characteristics of elastin mimetic peptide, they can repair themselves when damaged, so as to achieve long-term stable support effect and drug delivery (R. Chen et al., 2021). Poly(N-isopropylacrylamide), poly (N-isopropylacrylamide-co-polyacrylic acid), melamine cross-linked PEG, and some other materials containing amino groups are used for protein, peptide, and stem cell delivery (Bao et al., 2017; Mihalko et al., 2018; J. Tang et al., 2017). Take Poly(N-isopropylacrylamide) nanogels (FSN) as an example. The prevention and treatment of fibrosis require drugs to be released in a certain order and dynamically adjusted to improve the therapeutic effect and bioavailability of drugs. N-isopropylacrylamide (NIPAM) and N, N′-methylenebisacrylamide (BIS) are polymerized in a certain ratio, where NIPAM extends the main chain and provides hydrophilicity and BIS is used for cross-linking to form a mesh structure. The ratio of BIS affects the tightness of the crosslinking of the mesh structure, thereby affecting the degradation rate of the material at the damaged site, so as to achieve the gradual release effect (Figure 5A)(Mihalko et al., 2018). Due to the formation of scarring and fibrosis in the myocardial tissue, local electrical signal conduction is blocked, resulting in atrial and ventricular electrical signal rhythm disorders. In the long run, this will will affect the normal function of the heart, aggravate heart failure and lead to the reduction of left ventricular ejection fraction (He et al., 2020). Therefore, it is also necessary to increase the electrical conductivity of the gel. Polycarboxybetaine macromonomer polymerized with dithiothreitol (DTT) is an amphoteric material that can provide an environment for cell growth as well as conductive and antioxidant function (Liu Y et al., 2021). Artificial polymeric gel materials can achieve a variety of functionalities. In addition to the above gradual release and conductivity, they can also adjust the adhesion of materials (You et al., 2021), prolong the action time of materials, and protect deliverables from immune attack, etc. (Y. Chen et al., 2020; J. Tang et al., 2017)

**TABLE 2:** Synthetic hydrogel.

Category	Target	Materials	Features	References
Artificial synthesis	ECM	Poly(2-alkyl-2-oxazoline) (POx) derivatives of 2-ethyl-2-oxazoline and 2-butenyl-2-oxazoline	adhesion	You et al., 2021
		Cyclic peptide	Minimally invasive injectable	Carlini et al., 2019
		Polypyrrole-chitosan hydrogel (PPY-CHI)	Conductive	He et al., 2020
		Poly(N-isopropylacrylamide) nanogel (FSN)	Step by step release	Mihalko et al., 2018
		Polycarboxybetaine macromonomer and dithiothreitol (DTT) polymerized materials	Zwitterionic, conductive, antioxidant	Liu Y et al., 2021
	Cells	Thermosensitive poly (N-isopropylacrylamide-co-polyacrylic acid) or P(NIPAM-AA) nanogels	Low immunogenicity	(J. Tang et al., 2017)
		Melamine cross-linked PEG, cross-linked with thiol-modified hyaluronic acid	Soft and fatigue resistant	Bao et al., 2017
	Inflammation	Injectable water-based gel/mesoporous silica	Acid response	(Y. Li et al., 2021)

#### Injectable hydrogel

Hydrogels need to provide mechanical support in the myocardium, as well as continuous drug release and cardiomyocyte protection, requiring a certain mechanical strength and slow degradation of the hydrogel *in vivo*. However, in order to achieve injectable hydrogels, it must be in a state that can flow *in vitro*. Realizing the transition from the injectable state *in vitro* to the gel state *in vivo* requires that the material be able to undergo a phase transition based on the microenvironmental state, namely gelation (Diaz et al., 2021). According to the pathological process of myocardial fibrosis, the local microenvironment of fibrosis at the site of acute and chronic myocardial injury is different from normal tissue in terms of pH, ROS, and enzymes, as well as the aqueous environment *in vivo* and the normal physiological temperature of the organism are not the same as *in vitro*. Therefore, the material design can refer to the environment of fibrotic tissues in myocardium. It can be simply divided into chemical process gelation and biological process gelation.

#### Chemical process gelation

The chemical process gelation can respond to the aqueous environment *in vivo*, physiological temperature (37°C), and injection time. Gelation can also be achieved with the help of external conditions, such as light irradiation. Delivery system for co-delivery of miRNA by injectable hydrogel and mesoporous silica (Gel@MSN/miR-21-5p). Two types of interactions are utilized: i) hydrophobic interactions between cyclodextrins (CD) along aldehyde-based polyethylene glycol chains. ii) Schiff base reactions between amino groups from mesoporous silica and aldehyde-based polyethylene glycols (reaction of amino groups with aldehydes to form imine structures with the involvement of water), which cross-link to form network structures. The Schiff base reaction can also be used to achieve slow release of the delivery agent in response to the pH of the local inflammatory environment, to achieve polarization of macrophages to relieve inflammation and to increase blood supply to the local microenvironment (Y. Li et al., 2021). Responsive gelation can also be achieved using thermosensitive materials. Thermosensitive poly (N-isopropylacrylamide-co-polyacrylic acid) nanogels take advantage of the opposite rotation of the material under temperature change, and *in vitro* experiments have also been demonstrated that the material can gelate at 37°C to increase mechanical strength for delivery of hCSCs (J. Tang et al., 2017). In addition, modulating the gelation time of the material can enable *in vivo* injectability. For example, the Michael addition of thiols and alkenes was used in the previous study to construct polycarboxybetaine macromolecular monomers with dithiothreitol (DTT) polymerized materials (Liu Y et al., 2021). External aids can assist in gelation after gel injection. Using light irradiation, poly(2-alkyl-2-oxazoline) (POx) derivatives of 2-ethyl-2-oxazoline and 2-butenyl-2-oxazoline can be rapidly cured in less than 2 min. The tunable spatial structure of the synthetic gels allows their use for stem cell and protein/peptide delivery.

#### Bioprocess gelation

Decellularized extracellular matrix gels are a mature process in which decellularized extracellular matrix is obtained and digested by digestive enzymes to prepare an injectable extracellular matrix pre-gel, which is injected into the body and re-geled into the extracellular matrix state under physiological conditions (X. Wang et al., 2022; Wang X et al., 2021). Unlike the *in vitro* environment, the achievement of normal physiological functions of tissues involves the participation of a very large number of enzymes that are not present in the *in vitro* environment. As shown in Figure 5B, using a specific enzyme-substrate response design, the process of myocardial injury and fibrosis is accompanied by the increase of thrombin, and fibrinogen can coalesce into fibrin gel in the presence of thrombin. Fibrin gels based on this design involve heparin-loaded basic fibroblast growth factor-2 (FGF-2) and stromal cell growth factor-1α (SDF-1α) that are slowly released in the ECM to recruit stem cells and reestablish blood supply (Awada et al., 2017). The functionality of the enzyme can also be used to prepare cyclopeptide precursors, which are openedby the cleavage of metalloproteinase-2/9 (MMP-2/9) *in vivo* and repolymerized to form cross-linked protein gels (Figure 5C)(Carlini et al., 2019). The synthetic protein/peptide will be more biocompatible and able to be metabolically cleared. As with natural materials, protein gels can also be modified with proteins to obtain a material that is inherently beneficial for cardiac recovery. It can be used to deliver some cytokines, chemokines, and some small molecule drugs.

#### Hydrogel heart patch

Most of the materials used to make cardiac patches are biocompatible and highly elastic materials. Polymer materials such as PEG, graphene, HA, polylactic acid, polyamino esters, chitosan, and some protein materials such as silk protein and type I collagen have been used for the manufacture of cardiac patches (Feng et al., 2021; Melhem et al., 2017; Xie et al., 2022; Y. Yao et al., 2022).Patches is a kind of biomaterial, which can provide structural stability for damaged tissues under physiological conditions and maintain the stability of active substances such as cells and proteins (Melhem et al., 2017). Compared with injectable hydrogel, cardiac patch biomaterials adhere better to the heart and provide better drug release and support (Kim et al., 2019). However, the use of cardiac patches require invasive methods, such as surgical hand implantation. The inconvenience of use also limits the transfer of cardiac patch to clinical application.

#### Patch functionality

In stem cell therapy, the low retention rate of cell transplantation and the poor long-term survival of cells are the main problems of current cell therapy (J. Chen et al., 2018). As one of the optimal materials for stem cell therapy, patches provide an environment for stem cells to maintain normal function and longer retention time because of their better elasticity, cell viability and secretory activity (Kim et al., 2019). In one study, poly (ethylene glycol) dimethacrylate (PEGDMA) was designed with the aid of stereolithography (SLA) as a patch material with fixed pore sizes to accommodate to bone marrow-derived mesenchymal stem cells (MSCs) and maintain MSC cellular activity over time. Compared with patches loaded with MSCs without micropores, the ejection fraction and output per beat were improved (Melhem et al., 2017). Similarly, chitosan/silk proteins (CS/SF) multilayer modified electrostatically spun cellulose nanofiber patches enabled delivery of adipose-derived mesenchymal stem cells (AD-MSCs), thereby reducing local scarring, improving fibrotic status, decreasing cardiomyocyte apoptosis, and reconstructing blood supply in ECM (J. Chen et al., 2018).

Synthetic polymeric materials usually allow for some functionalization using the chemical structure of the polymer. In the early stages after acute myocardial injury, with the death of apoptotic cardiomyocytes, cell rupture increases the level of ROS in the local microenvironment. The ROS response can be achieved by inserting the structure of thioacetal ketone (PTK) and poly (propylene fumarate) (PPF) into elastic polyurethane (PFTU). In combination with pro-angiogenic Arg-Glu-Asp-Val (REDV) peptide and Rosuvastatin (PRR), excess ROS are exhausted, and the intelligent on-demand release of proteins and drugs is realized depending on the severity of injury, followed by REDV and PRR action during fibrosis to inhibit the fibrotic process. This delivery platform enables dynamic delivery of therapeutic components to target the pathological process of myocardial fibrosis (Xie et al., 2022; Y. Yao et al., 2022)

Cardiac patches make an ideal material for maintaining normal cellular function and are very suitable for therapeutic strategies that require long-term maintenance (Liu M et al., 2021). However, due to the limitations of its preparation cost, material preservation and implantation methods in its application, the use of patches is still limited. At the same time, most of the functions that can be achieved by the patch can also be achieved by injectable hydrogels. It is undeniable that patches has their own advantages, however, the clinical translation potential of cardiac patches has been controversial (K. Huang et al., 2020; J. Yao et al., 2021)

#### Release of the cargo

Natural material hydrogels are mainly loaded in the form of physical encapsulation such as cytokines, chemokines, enzymes and their inhibitors, and encapsulated cells. On the other hand, synthetic hydrogels, can also release small molecule drugs through chemical bonding reaction. Because some cytokines, such as basic fibroblast growth factor (FGF-2) can promote vascular growth, the transient release of a large number of cytokines has the possibility of inducing tumors. Therefore, when using this active molecule as a therapeutic agent, the release rate must be controlled (C. Fan et al., 2019). The polymerization tightness of the hydrogel can be modulated to accelerate the release rate of the drug, or it can be combined with natural macromolecules to achieve controlled release. In one study, FGF-2 and stromal cell growth factor (SDF-1α) were encapsulated in a cohesive layer of heparin, With the degradation of the heparin adhesive layer, FGF-2 is slowly released to promote angiogenesis, SDF-1 α Mesenchymal stem cells are recruited to achieve a variety of effects, such as inhibiting ECM degradation, rebuilding blood supply, and reducing fibrosis (Awada et al., 2017). Hydrogel modification by itself still has limitations, while hydrogel combined with nanoparticle delivery platform can achieve a variety of delivery functions. FGF-2 was designed as nanoparticles covalently linked by MMP-2/9 cleavable peptide plalga (TIMP) and glutathione S-transferase (GST), forming a bifunctional Mi responsive hydrogel The binding between GST and reduced glutathione (GSH) is utilized to increase drug delivery. While TIMP responds to MMP-2/9 cleavage to release the drug,it also inhibited MMP-2/9 overexpressed after myocardial injury (TIMP is a competitive substrate for MMPs)(C. Fan et al., 2019).Compared with other nano materials, hydrogels have irreplaceable advantages in of biocompatibility, mechanical support, and cell protection. However, using hydrogels alone cannot solve the problem of single type drug delivery and difficult functionalization. Therefore, combining the advantages of a variety of biological nano materials, the development of cardiac drug delivery platform is a promising direction.

Currently, gel material is the “best material” for cardiomyocyte damage repair. Compared with nanoparticles, patches, etc., injectable hydrogels can mimic the normal functional environment of cardiomyocytes to the maximum extent. As a drug delivery material, hydrogel itself has certain functionalities, such as regulation of inflammation, promotion of new blood vessel growth, softening of cardiac tissue, and so on. With the use of cytokines, chemokines, etc., it is expected to restore the normal finishing state of the ECM. In addition, natural and synthetic gels have their own advantages Natural hydrogels still have some unknown mechanisms to achieve better biological adaptability and good recovery of damaged tissues, while synthetic hydrogels can confer more functionalization. In addition, hydrogels can be combined with other organic and inorganic nanomaterials to achieve more intelligent treatment targeting the local environment of cardiomyocyte injury. As a traditional biomaterial, hydrogels still have great potential for development and improvement. It can be applied to some injurious diseases such as myocardial injury, joint injury, and so on Injectable hydrogels should be used as a base platform for developing more functional biomaterials, rather than an independent system.

### Nanoparticles

The process of myocardial fibrosis involves several pathological mechanisms. Generally the use of inhibitors of inflammation and immunity, stimulation of local blood supply reconstruction, inhibition of the myofibroblast growth process and supplementation of stem cells have positive effects on fibrosis (Vazir, Fox, Westaby, Evans, and Westaby, 2019). Currently, nanoparticles drugs in cardiovascular disease have gradually changed from traditional small molecules particle delivery to proteins, nucleic acids, and other biological macromolecules (Bejerano, Etzion, Elyagon, Etzion, and Cohen, 2018; Hong et al., 2020; Kwon et al., 2021; S. Liu et al., 2020; Mihalko et al., 2018; Wang Q et al., 2021; X. Zhang et al., 2022). Taking the delivery of miRs as an example, miR-1, 133, 208, and 499 are capable of cardiac reprogramming, and the conversion of fibroblasts into induced cardiomyocyte-like cells is expected to be a new approach for the treatment of myocardial fibrosis. However, microRNAs need to be delivered to fibrotic site is required to achieve these effects Nanoparticles can change the pharmacological properties of drugs, thus enabling drugs with systemic toxicity and non-targetable characteristics to be used in myocardial fibrosis and are expected to be new drugs for clinical use (Wang Q et al., 2021).

Nanoparticles, an emerging drug delivery system, hold the promise of solving a very large number of challenges in drug delivery for cardiovascular diseases. However, compared with the field of oncology, the field of cardiovascular nanomedicine has only been initially developed in recent years (Skourtis et al., 2020). In cardiovascular disease, due to high velocity blood supply, nanoparticles must be highly stable and need to evade immunophagocytosis (Gupta et al., 2019). Therefore, several nanocarriers, have been investigated and designed, including polymers, liposomes, and inorganic nanoparticles. Polymeric nanoparticles and liposomal nanoparticles are more common, which can generally stabilize circulation in the blood stream and prolong the retention time of drugs in the body (Bertrand, Wu, Xu, Kamaly, and Farokhzad, 2014). Passive targeting can be achieved by surface charge characteristics, size, and shape of nanoparticles, or by surface groups or protein modification, cell membrane wrapping, etc. (Table 3)(Mihalko et al., 2018; Takahama et al., 2009; H. Yang et al., 2019)

**TABLE 3:** Nanocarriers for myocardial fibrosis.

Category	Material	Cargo	Effects	References
Polymer	Hyaluronic acid sulfate	miRNA	Anti-inflammatory	Passaro et al., 2021
	Chitosan (CS) @ sodium tripolyphosphate (TPP)	Ginsenoside Rb3	Improvement of fibrosis	(Y. Zhang et al., 2021)
	Polylactic acid glycolic acid (PLGA)	Secretory factor (SF)/copper complex	Blood supplies	(X. Zhang et al., 2022)
	Branched poly (β-amino ester)	siRNA	Anti-inflammatory	(X. Wang et al., 2020)
	Fibrin specific poly (N-isopropylacrylamide) nano gel (FSN)	Tissue fibrinogen activator (tPA)/cell contractility inhibitor (Y-27632)	Blood supplies/Improvement of fibrosis	Mihalko et al., 2018
	Poly lysine (DGL)	miRNA	Blood supplies	Hong et al., 2020
Inorganic nanoparticles	Mesoporous silica	miRNA	Reduction of fibroblasts	Wang Q et al., 2021
	Calcium carbonate nanoparticles	Colchicine	Anti-inflammatory	Wang L et al., 2021
	Iron oxide/silica shell	Vesicle capture *in situ*	Restoration of cardiac function	(S. Liu et al., 2020)
	Graphene oxide		Anti-inflammatory	Han et al., 2018
Organic inorganic hybrid	Mesoporous silica	Gene transfected mesenchymal stem cells	Protection of cardiac muscle cells	Zhu et al., 2016
Liposomes	MI antigen and rapamycin (L-Ag/R) in liposomal nanoparticles	MI antigen and rapamycin (L-Ag/R) in liposomal nanoparticles	Induction of immune tolerance	Kwon et al., 2021
	DSPE	FK506	Immunosuppression	Okuda et al., 2016

#### Polymeric nanoparticles

Polymer nanoparticles are usually made of biodegradable materials, such as chitosan (CS), polylactic acid-ethanolic acid (PLGA), HA, amino acids (e.g., lysine), some amino ester materials, etc. It can be used for the synthesis of polymer nanoparticles (Hong et al., 2020; Mihalko et al., 2018; X. Wang et al., 2020; X. Zhang et al., 2022; Y. Zhang et al., 2021). By utilizing the hydrophilic, charge properties, conjugation, or covalent linkage of the material, polymers can load drugs in a variety of ways, so that drugs can be released according to the characteristics of local microenvironment (such as pH, ROS, temperature, etc.) t (e.g., pH, reactive oxygen species, temperature, etc.)(Li Y et al., 2020; X. Wang et al., 2020).

**FIGURE 6:**
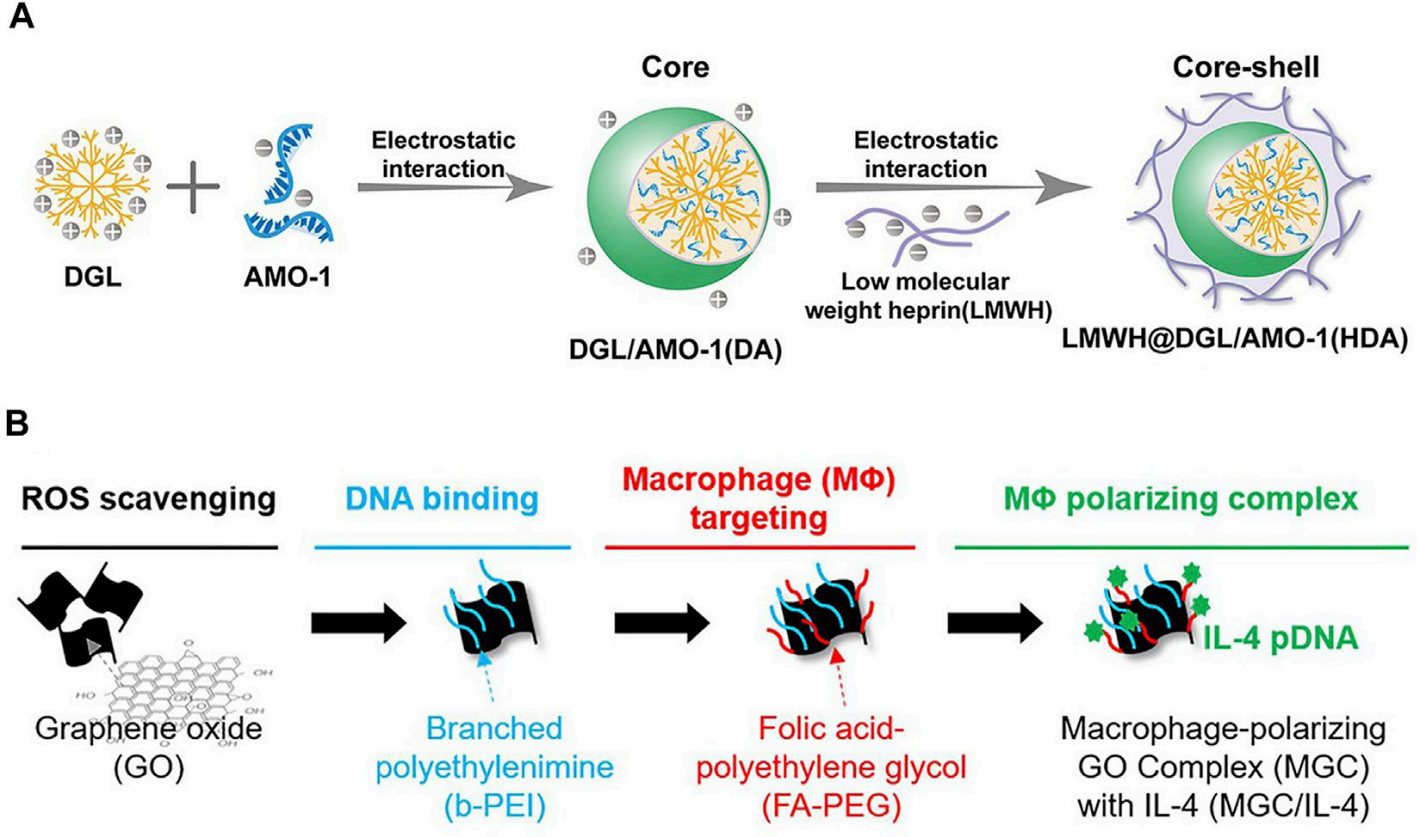
**(A)** Low molecular weight heparin (LMWH)-coated polylysine (DGL) For local microthrombosis(Hong et al., 2020). **(B)** PEG-modified graphene oxide for achieving M2-type macrophage polarization(Han et al., 2018).

Based on this, a fibrin-specific poly(N-isopropylacrylamide) nanogels (FSNs) loaded with tissue fibrinogen activator (tPA)/cellular contractility inhibitor (Y-27632) have been developed using hydrophobic properties. FSNs can specifically recognize fibrin in damaged cardiac sites, enabling targeting and improve blood supply at the same time. They address the need to re-establish blood supply in the local microenvironment and inhibit myocardial fibrosis after I/R injury (Mihalko et al., 2018). The surface charge properties of polymers are determined by the specific groups on the surface. Nanoparticles with a large amount of amino groups present tend to carry a positive charge at physiological pH. On the contrary, polymer nanoparticles usually carry a large amount of carboxyl groups a partial negative charge at physiological pH. Dendritic polylysine (DGL), which can be used to carry the internal wrapping ofAMO-1 (a miRNA that exhibits negative electrical properties and is used to improve myocardial fibrosis) while the outer layer is attached to the negatively charged low molecular weight liver (LMWH). Reduces microthrombus formation in microvessels, delivers AMO-1 to cardiomyocytes through microvessels, and enhances uptake by cardiomyocytes in the infarct area (Figure 6A)(Hong et al., 2020). Nanoparticles use polymeric nanoparticles to achieve responsive release according to the local microenvironment of damaged cardiomyocytes. A branched poly(β-amino ester) with a built-in redox-responsive structural domain (achieved through disulfide bonds) that provides effective siRNA affinity through a multi-branched structure and is degraded by intracellular reduced glutathione (GSH) into small fragments upon internalization into rat cardiac microvascular endothelial cells (RCMECs) for on-demand release (X. Wang et al., 2020).In addition, nanoparticles can be degraded into small fragments through pH reaction, ROS reaction photothermal, and magnetic properties, which can be used as a nano-delivery platform to target cardiac fibrosis .

#### Inorganic nanoparticles

Compared with organic synthetic and natural nanoparticles, inorganic nanoparticles have some unique properties such as magnetic properties, long-term stability, etc. (Sharma, Filip, Zboril, and Varma, 2015). Inorganic nanoparticles are usually synthesized using some metallic materials, such as gold, silver, iron, titanium, and some lanthanide elements (Luther et al., 2020). Nanoparticles are composed of iron oxide and silica dioxide, silica dioxide binds to both antibodies through hydrazone bonds and captures extracellular vesicles through the CD63 antigen on the surface of the extracellular vesicles (EV). The damaged myocardial site is acidic at pH and the hydrazone bond is broken to release EVs, which enables the capture and release of exosomes under the control of an external magnetic field (S. Liu et al., 2020). Silicon dioxide, calcium carbonate, and carbon materials can also be used to build nanoparticles (Zhu et al., 2016; Luther et al., 2020). The mesoporous silica-rich hollow structure is used to load miRNAs, and cell membranes modified with FH peptide (a peptide with high affinity for tendin-C (TN-C) produced by cardiac fibroblasts) with active targeting ability are used to target myofibroblasts for direct cardiac cell reprogramming and transformation of fibroblasts into induced cardiomyocyte-like cells (Wang Q et al., 2021). Inorganic nanoparticles could perform a variety of functions. However, inorganic nanoparticles are widely distributed in the organism and are not easy to remove, which raises concerns about the potential risks of accumulation of inorganic nanoparticles in the body, as well as the risks of toxicity and teratogenicity (Sharma et al., 2015; Soenen, Parak, Rejman, and Manshian, 2015; Medici, Peana, Pelucelli, and Zoroddu, 2021). For its potential risks, preliminary material screening can be carried out through high-throughput screening combined with toxicological experiments (Nel et al., 2013). Modification of the surface of nanomaterials can also reduce the toxicity of the material to some extent, such as PEG modification, which will reduce the ability to adhere to the surface of particles and reduces phagocytosis (Figure 6B)(Han et al., 2018; Heuer-Jungemann et al., 2019; C. Yang, Tian, and Li, 2016). Although the toxicity of inorganic nanoparticles has been studied, the metabolic clearance of inorganic materials in the body has been an unsolvable problem from the beginning, and the aggregation of inorganic materials in the body is also the inevitable result of the use of inorganic nanoparticles; the clinical application of inorganic nanoparticles in myocardial injury related diseases still has a long way to go.

#### Liposome nanoparticles

Liposome nanoparticles are composed of phospholipid bilayers with a biofilm-like structure, which can be used to load hydrophilic and hydrophobic drugs due to their amphiphilic structural properties. We can change the head and tail structure of liposomes to realize the functionalization of liposomes, such as prolonging the circulation time of liposomes, achieving active targeting, targeting microenvironmental response, and multifunctional liposomes (Y. Fan, Marioli, and Zhang, 2021; M. Li et al., 2019). Liposomes are commonly used to provide immunosuppressants in myocardial injury diseases. Targeting dendritic cells with liposomes and immunosuppressants encapsulated with myocardial injury-associated antigens, can induce specific immune tolerance and inhibit the transformation of cardiac fibroblasts stimulated by chronic inflammatory response into muscle fibroblasts Dendritic cells can “nest” at the site of myocardial injury, so liposome nanoparticles can target dendritic cells by subcutaneous injections (Kwon et al., 2021). However, due to its own stability, liposomes have low bioavailability of direct cardiovascular targeting, and surface optimization is again needed to increase cycle time and targeting capacity (Takahama et al., 2009; Ferreira et al., 2017). There are few studies on liposome delivery of drugs to cardiomyocytes, but liposome nanoparticles have advantages due to their cell membrane-like structure and good biocompatibility. Liposomal nanoparticles combined with the advantages of other materials may have a broad prospects in cardiac drug delivery.

### Spray

Curable-applied nano-spray is a new kind of cardiac nano-biomaterial, which can enable minimally invasive drug delivery and long-term retention of patch-like structures in the cardiac region. Fibrinogen-loaded exosomes (EVS) were used as the spray material and thrombospondin as the sequestering agent to form a long-term adhesive exosome delivery system in the form of spray in the infarcted region. In a mouse model of acute MI, EXOS improved cardiac function and reduced fibrosis, and promoted endogenous angiomyogenesis in the post-injury heart. (J. Yao et al., 2021). A mixture of gelatin methacrylate (GelMA) precursor and photoinitiator were also used for spraying into the infarcted area, used visible light irradiation to form a gel (GelMA) that wraps around the heart surface and slowly releases EVs through gel diffusion and enzymatic degradation, resulted in a better effect of EVs (J. Tang et al., 2022). Cardiac sprays are a good solution to the problems of hydrogels and patches, i.e., retention time, supportive surgical implantation trauma, etc. However, it is also limited by production conditions, complexity of use. Today’s sprays for cardiac use need to be atomized and transported active substances together, which also limits the composition of active substances that can be delivered, generally proteins, exosomes, etc., while nucleic acids, stem cells, etc., are not applicable.

## Conclusion and outlook

With the continuous exploration of the relationship between fibrosis and inflammation mechanical stress, and electrical signals as well as the continuous development of biomaterials has been progressively explored. The combination of biomaterials and myocardial fibrosis diseases is gradually being widely explored, so the therapeutic strategy for myocardial fibrosis is changing from the traditional blocking of fibrosis to the restoration of cardiac function. Using biomaterial delivery systems to transport the delivery of some biological agents can adequately address the problems of difficult use, low bioavailability, and easy deactivation of biological agents. MicroRNA, cytokine and chemokine therapies, stem cell therapies, and exosome therapies can achieve a certain degree of cardiomyocyte protection and restoration of cardiac function, and this shift offers a new strategy for the treatment of myocardial fibrosis. Inflammation and immunity are also inextricably linked to fibrosis, and biomaterials combined with inflammatory processes as well as immune regulation also demonstrate a powerful role in myocardial fibrosis. The role of biomaterials as an adjunct to therapy broadens the research horizon and improves the feasibility of many therapeutic strategies. Unfortunately, there are still many problems in the current strategies for the treatment of myocardial fibrosis. Because myocardial fibrosis is inevitably accompanied by the loss of cardiomyocytes, the loss of cardiomyocytes will cause permanent damage to the heart tissue, resulting in “insufficient power” of the heart. Which is the main problem in cardiac diseases related to myocardial fibrosis, However, popular theories believe that this process is irreversible. Thus, despite the variety of current therapeutic strategies, we still have not resolved the critical issue of myocardial fibrosis or even fibrosis in other organs. Moreover, the treatment achieved through short-term strategies rarely produce long-term practical results, which limits the clinical translation of biomaterials for myocardial fibrosis research. Therefore, promoting the clinical translation of existing research findings that preserve myocardial function after injury and maximizing the preservation of cardiac function with existing treatment strategies is the best strategy to benefit patients. Patients also expect to see new therapeutic approaches and new drug delivery platforms in the future.

## Data Availability

The original contributions presented in the study are included in the article/Supplementary Material, further inquiries can be directed to the corresponding authors.
